# Multi-omics analysis in inclusion body myositis identifies mir-16 responsible for HLA overexpression

**DOI:** 10.1186/s13023-024-03526-x

**Published:** 2025-01-15

**Authors:** Daphne Wijnbergen, Mridul Johari, Ozan Ozisik, Peter A.C. ‘t Hoen, Friederike Ehrhart, Anaïs Baudot, Chris T. Evelo, Bjarne Udd, Marco Roos, Eleni Mina

**Affiliations:** 1 Department of Human Genetics, Leiden University Medical Center, Leiden, The Netherlands; 2 Harry Perkins Institute of Medical Research, Centre for Medical Research, University of Western Australia, Nedlands, WA Australia; 3 Folkhälsen Research Center, Helsinki, Finland; 4 Department of Medical and Clinical Genetics, Medicum, University of Helsinki, Helsinki, Finland; 5 Université Paris Cité, INSERM U976, Paris, France; 6 Department of Medical BioSciences, Radboud university medical center, Nijmegen, The Netherlands; 7 Department of Bioinformatics - BiGCaT, NUTRIM/MHeNs, Maastricht University, Maastricht, The Netherlands; 8 Aix Marseille University, INSERM, MMG, Marseille, France; 9 CNRS, Marseille, France; 10 Barcelona Supercomputing Centre, Barcelona, Spain; 11 Department of Bioinformatics - BiGCaT, NUTRIM, Maastricht University, Maastricht, The Netherlands; 12 Tampere Neuromuscular Center, University Hospital, Tampere, Finland

**Keywords:** Inclusion body myositis, Multi-omics, Transcriptomics, Genomics, Network analysis, Active subnetwork identification, Rare diseases, Data integration, Multiplex network

## Abstract

**Background:**

Inclusion Body Myositis is an acquired muscle disease. Its pathogenesis is unclear due to the co-existence of inflammation, muscle degeneration and mitochondrial dysfunction. We aimed to provide a more advanced understanding of the disease by combining multi-omics analysis with prior knowledge. We applied molecular subnetwork identification to find highly interconnected subnetworks with a high degree of change in Inclusion Body Myositis. These could be used as hypotheses for potential pathomechanisms and biomarkers that are implicated in this disease.

**Results:**

Our multi-omics analysis resulted in five subnetworks that exhibit changes in multiple omics layers. These subnetworks are related to antigen processing and presentation, chemokine-mediated signaling, immune response-signal transduction, rRNA processing, and mRNA splicing. An interesting finding is that the antigen processing and presentation subnetwork links the underexpressed miR-16-5p to overexpressed HLA genes by negative expression correlation. In addition, the rRNA processing subnetwork contains the *RPS18* gene, which is not differentially expressed, but has significant variant association. The *RPS18* gene could potentially play a role in the underexpression of the genes involved in 18 S ribosomal RNA processing, which it is highly connected to.

**Conclusions:**

Our analysis highlights the importance of interrogating multiple omics to enhance knowledge discovery in rare diseases. We report five subnetworks that can provide additional insights into the molecular pathogenesis of Inclusion Body Myositis. Our analytical workflow can be reused as a method to study disease mechanisms involved in other diseases when multiple omics datasets are available.

**Supplementary Information:**

The online version contains supplementary material available at 10.1186/s13023-024-03526-x.

## Background

Inclusion body Myositis (IBM) is a rare, acquired muscle disease with a prevalence ranging from 24.8 to 45.6 per million people [1], but the most common muscle disease with onset after age 50 [2]. The molecular pathogenesis of IBM has consistently been of high interest due to the unexplained combination of inflammatory changes, degenerative features, and mitochondrial abnormalities in the muscle tissue [3, 4]. One hypothesis suggests that autoimmunity drives protein aggregation, resulting in high interferon-gamma and cytotoxic T-cell responses [5]. Endomysial infiltration of CD8 + T cells in IBM muscles and the reported clonal expansion of these T cells in the blood and muscles of IBM patients suggest the presence of unknown antigens [6]. Currently, the links between these antigens, protein aggregate pathology, and the immune response are poorly understood. Identifying these links could contribute to understanding the disease pathomechanisms and thereby to the development of more effective diagnosis and treatment.

The integrated analysis of multiple types of omics datasets (multi-omics analysis) may provide new insights into potential disease causing mechanisms as well as knowledge about their interplay. A single omics layer often provides information about a single aspect of one type of molecule. For instance, in processed transcriptomics datasets, the abundance of the mRNA is usually the only information provided. Integrating this data with other omics, like genomics and microRNA (miRNA) transcriptomics, can provide a better picture of the molecular state of cells in the disease. In addition, a multi-omics approach can increase the statistical power of analyses, even when the number of available patient samples is limited due to the rare occurrence of the disease [7]. Finally, multi-omics approaches can give more insight into the flow of information in the disease, for example, from genetic factors to their consequences [8].

In biological research, there is an abundance of knowledge available from previous experiments and research, such as protein-protein interactions and pathway information. This prior knowledge can be used in research, and benefit the study of rare diseases by allowing more and diverse information to be used in the analysis, despite the limited number of patient samples. To fully exploit prior knowledge and multi-omics data analysis, we combined these two to increase the amount of information available. This has several benefits [9] (i) focusing the analysis on the results that are more likely to be biologically relevant, (ii) deprioritizing spurious results arising from noise instead of biological signals, since they are less likely to be associated with prior knowledge, and (iii) providing extra knowledge and data together with the results, which supports the formulation of hypotheses after analysis.

In this work, we performed an integrative multi-omics data analysis with prior knowledge to investigate mechanisms that are disrupted in IBM. We created a large-scale network combining different types of interactions involving genes/proteins and miRNAs. Using an active subnetwork identification algorithm, we identified several subnetworks that were highly relevant for IBM and reflected processes that are already known to be affected in IBM, but also some novel ones.

## Methods

### Workflow

**Fig. 1:**
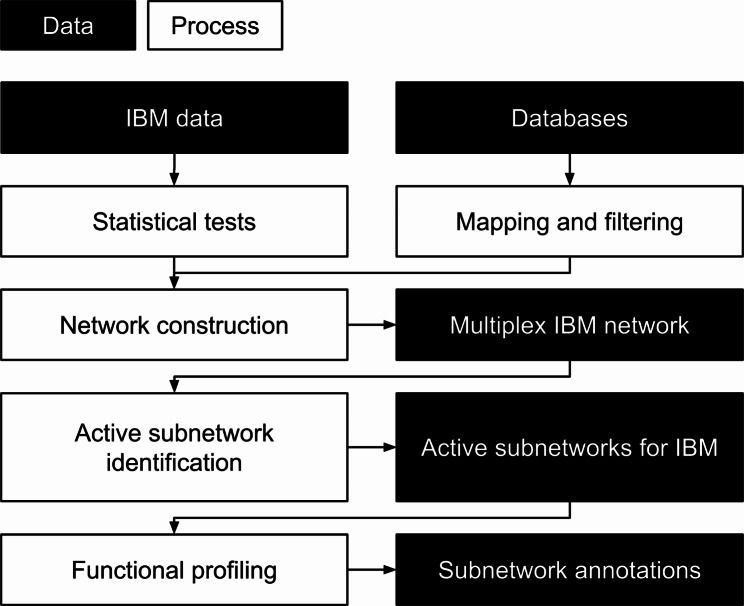
The overview of the complete workflow. The IBM data is combined with knowledge from databases in order to construct a network. Active subnetwork identification is applied on this network to find active subnetworks. These subnetworks are subsequently annotated with Gene Ontology annotation

We implemented a workflow that consists of multiple steps, namely differential expression testing, burden testing, network construction, active subnetwork identification and functional profiling. An overview of the workflow is shown in Fig. 1. To make the workflow more Findable, Accessible, Interoperable and Reusable (FAIR) [10], we made it available on WorkflowHub [11]. The workflow is developed in Common Workflow Language (CWL) [12], which ensures the scripts always run the same way when reused. It also allows metadata to be embedded in the inputs, outputs and steps of the workflow for the purpose of findability and reusability. Finally, we made a Docker container [13], and attached it to the workflow to ensure our computational environment is reproduced upon reuse.

### RNA-Seq differential expression analysis

We obtained gene expression count datasets (both mRNA and miRNA) from the Gene Expression Omnibus database (GEO) using the GSE151758 accession code, which have samples for IBM patients and amputee controls. This dataset was generated by Johari et al. [14] using short-read polyA + RNA sequencing from muscle biopsy tissues. To ensure that we have an integrated network in which the miRNA and mRNA results reflect the same biological changes, we used only the eighteen IBM and nine control samples which had both mRNA and miRNA data available.

We repeated the differential gene expression analysis (with the original script) that was performed by Johari et al. [14] in R (version 4.0.5) [15, 16], using Bioconductor (version 1.30.10) [17] and DESeq2 (version 1.30.1) [18]. With DESeq2, the raw data was transformed to be approximately homoskedastic and normalized for factor and library size. The data was then fitted to a Negative Binomial Generalized Linear Model and tested for differential expression between the IBM and amputee cohorts using the Wald test (additional file 1 and 2).

### Exome sequencing variant burden test

We expanded upon our previously published Finnish IBM cohort [19], bringing the total to 81 Finnish IBM patients. For the 51 additional individuals, we produced exome sequencing data as described previously [19]. This dataset includes the 18 Finnish IBM patients for whom we also have mRNA and miRNA data available. We used Finnish controls (*n* = 99) from 1000 Genomes project and downloaded the exome sequencing datasets for the same. We then generated genotypes in VCF format for both cases and controls. To increase the statistical power of the analysis, we performed a rare variant burden test using the “RVTESTS” software (version 2.1.0) [20, 21], suitable for testing rare variants with different directions of effects. We used RVTESTS with the following parameters: Burden = CMC (Combined Multivariate and Collapsing), Kernel = SKAT (Sequence Kernel Association Test), and Variable threshold model by permutation (price). We used the resulting *p*-values from the SKAT test in subsequent analysis.

### IBM multi-omics multiplex network construction

**Fig. 2:**
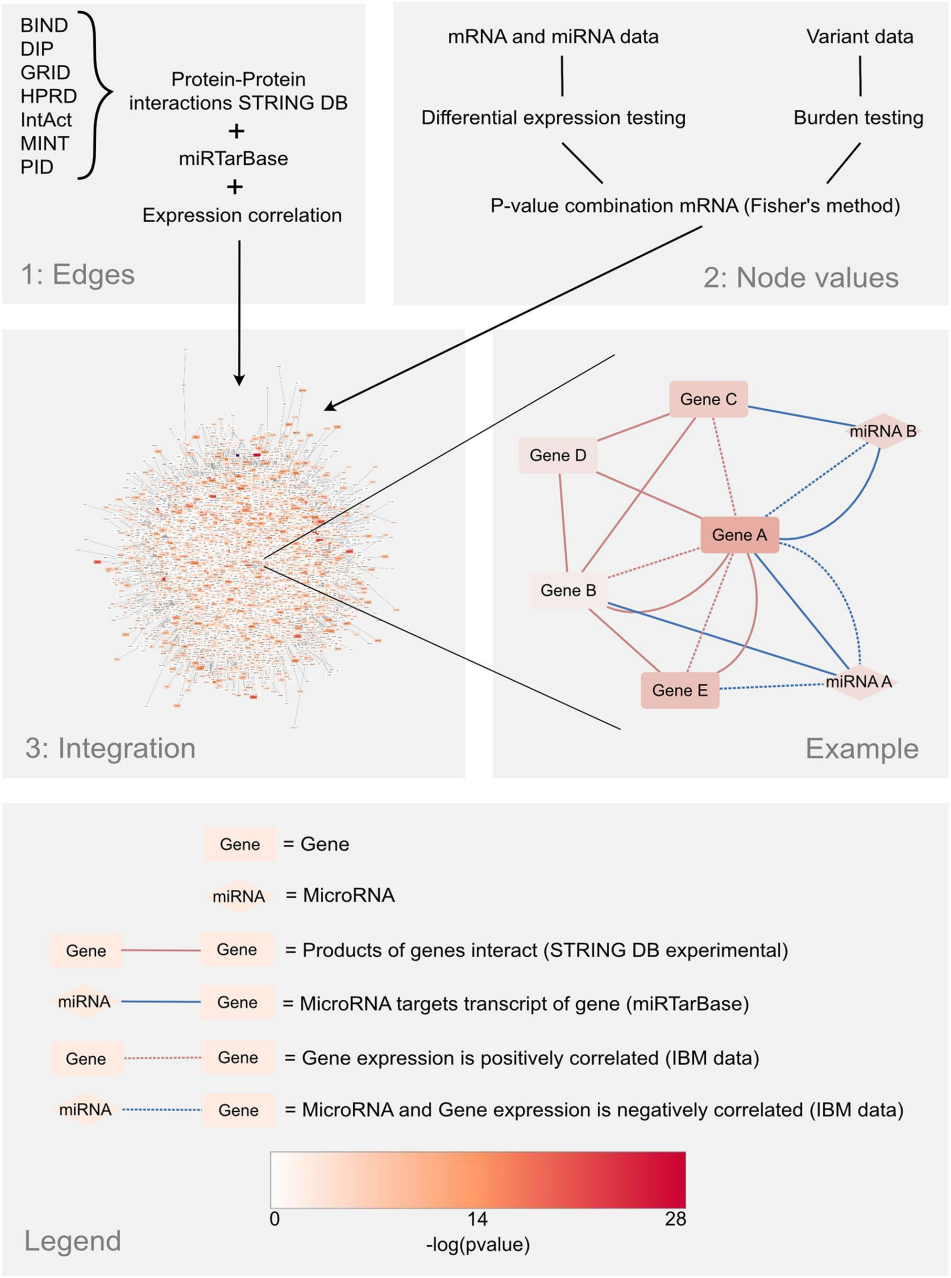
The workflow for the network creation. Step 1: Interaction data is downloaded from STRING DB and miRTarBase. Expression correlation is calculated from the IBM expression datasets. These are mapped and combined into a database layer, and a correlation layer. Step 2: Differential gene expression testing is applied to the mRNA and miRNA data. Burden testing is applied to the exome sequencing data. In the case of mRNAs, the two *p*-values are combined into one. Step 3: The node values are overlaid on top of the network created from the edges

We created a multiplex multi-omics IBM network comprising two layers (Fig. 2). The first layer is composed of protein-protein interactions that have an experimental evidence score of at least 0.200 (to filter out low-confidence interactions) in STRING [22] (version 11.0) and miRNA-mRNA target pairs from miRTarBase [23] (version 8.0). The second layer comprises mRNA-mRNA and miRNA-mRNA pairs, with their biweight midcorrelation calculated from the normalized transcriptomics data using the equation defined in [24]. In our calculation, only samples that had data in both transcriptomics datasets, and only genes with expression in more than half of the samples were used. In order to capture the correlations (and directions of correlations) with the most biologically informative information, the correlations were transformed to a binary form by setting a threshold of > 0.7 for mRNA-mRNA pairs and a more relaxed threshold of <-0.5 for miRNA-mRNA pairs (lower mean correlation). In the resulting multiplex multi-omics network, a node represents either a miRNA or a gene. Note that the gene node corresponds to both the mRNA and the protein. Regarding the miRNA nodes, we assigned a *p*-value on each node based on their differential gene expression. Similarly, for each gene node we assigned a combined *p*-value using Fisher’s method based on the differential gene expression and the variant burden of each particular gene. Note that this *p*-value is only valid for prioritization and not for statistical inference, since different null hypotheses are combined.

Finally, to connect everything in the network, we used BridgeDB [25] and miRBaseConverter [26] to map the identifiers of STRING and miRTarBase to the identifiers of the transcriptome and exome datasets. Specifically, we mapped NCBI gene identifiers to Ensembl identifiers, and mapped miRNA names to miRBase accessions. We removed isolated nodes that were not connected to the largest connected component of the network, because we are using an iterative analysis method that traverses the network. The resulting network consists of 17,405 nodes and 3,353,996 edges of different types (Table 1).

**Table 1:** Summary statistics for the multiplex network

Statistic	Count
Number of nodes	17,405
Number of gene nodes	15,574
Number of miRNA nodes	1,831
Number of genes with variant burden (padj < 0.05)	209
Number of mRNA differentially expressed (padj < 0.05)	8215
Number of miRNA differentially expressed (padj < 0.05)	628
Number of edges	3,353,996
Number of STRING edges	300,536
Number of miRTarBase edges	153,694
Number of mRNA-mRNA correlation edges	2,157,070
Number of miRNA-mRNA correlation edges	742,696

### Active subnetwork identification

We used MOGAMUN [27] to identify highly interconnected subnetworks that have a high degree of biological significance (active subnetworks) using default parameters. Thirty parallel runs were performed with 500 generations of optimization each. Since subnetworks are allowed to overlap, they were merged during post processing in MOGAMUN. In this process, the maximum number of nodes per subnetwork was increased to 200, and the Jaccard Index threshold was lowered to 0.2, in order to obtain subnetworks that are highly distinctive. We performed functional profiling of each resulting subnetwork with Gene Ontology Biological Processes terms using the g: GOSt function in the gProfiler2 R package (version 2.0) [28]. We also calculated the correlation of each subnetwork’s first principal component with the estimation of several cell types as estimated by Johari et al. [14, 29, 30].

## Results

**Fig. 3:**
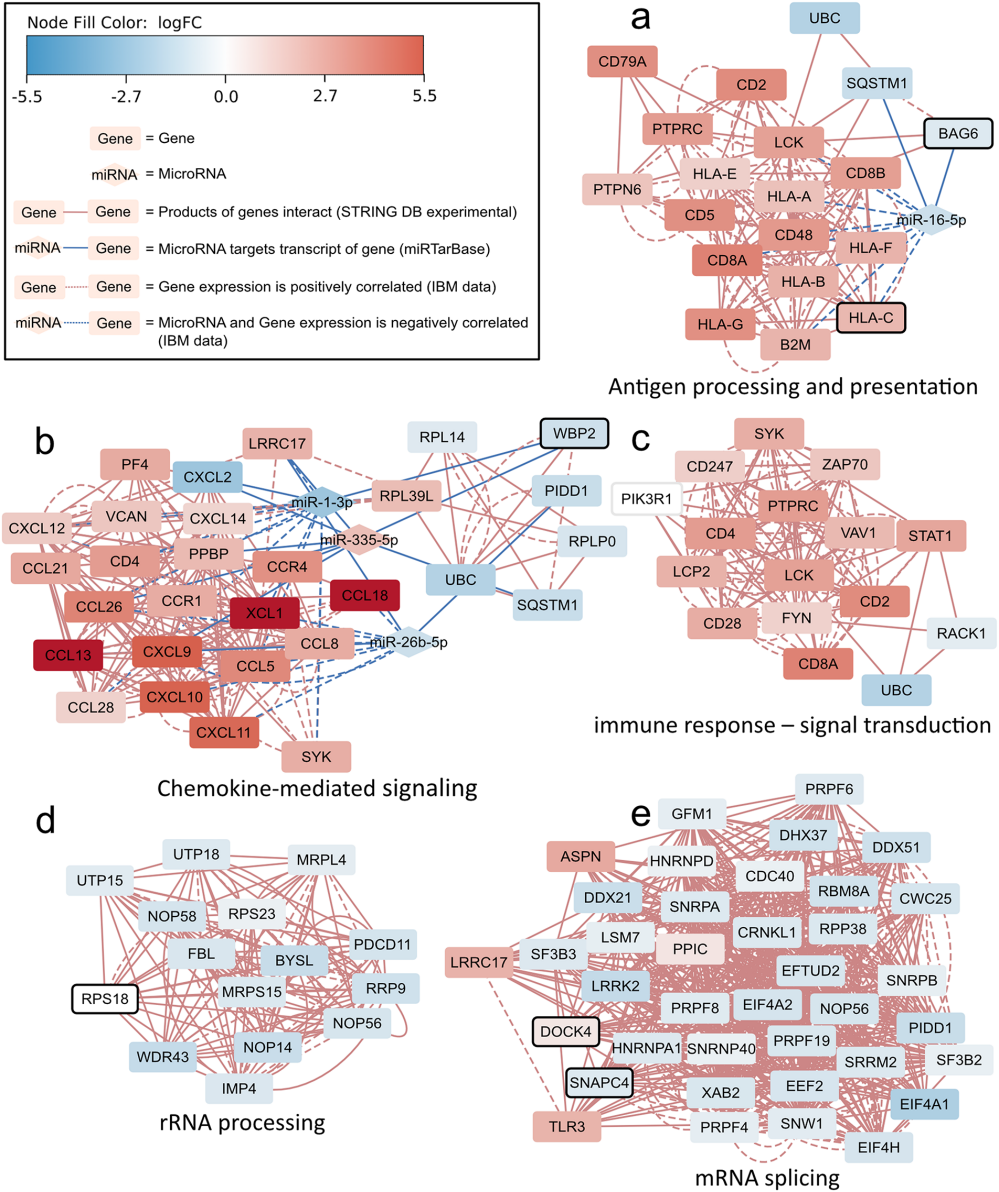
The five subnetworks that were identified by the active subnetwork identification algorithm together with their top-scoring GO annotations. Node shapes indicate the type of each entity. The gene and miRNA expression fold change is shown using color (red indicates upregulation; dark red indicates a fold change > 5.5; blue indicates downregulation; white indicates no change). Variant burden significance is shown with a solid line around a node. The different types of an edge are depicted with the line type

In order to elucidate IBM disease mechanisms from multi-omics data, we applied active subnetwork identification [27] on our IBM network. This resulted in five distinct subnetworks which we labeled according to their top GO terms: “Antigen processing and presentation”, “Chemokine-mediated signaling”, “Immune response – signal transduction”, “rRNA processing”, and “mRNA splicing”, respectively (Fig. 3). These subnetworks are optimized based on both the density of interactions and the scores (*p*-values) of the nodes. All *p*-values, correlations and fold changes in these subnetworks are available in additional file 3 and 4.

The “Antigen processing and presentation” subnetwork (Fig. 3a) contains 20 nodes, of which 19 are genes, and one is a miRNA. These 20 nodes are all significantly differentially expressed (padj < 0.05) and two of those *(HLA-C* and *BAG6*) also have a significant variant burden (padj = 0.013 and padj = 0.008, respectively). Six nodes are overexpressed HLA genes (*HLA-A*, *HLA-B*, *HLA-C*, *HLA-E*, *HLA-F*, and *HLA-G*). In previous studies, their overexpression was consistent in IBM and other inflammatory myopathies [14, 31]. This subnetwork also contains several overexpressed CD genes, namely *CD2*, *CD5*, *CD48*, *CD79A*, *CD8A*, and *CD8B*, which are markers of Leukocytes [32]. *LCK* was another overexpressed gene in this subnetwork, previously proposed as an apoptosis regulator involved in IBM [14]. Besides protein-coding genes, this subnetwork contains the miRNA miR-16-5p, linked to the *HLA-A*, *HLA-B*, and *HLA-C* genes, with negative correlation coefficients ranging from − 0.51 to -0.58. Finally, this subnetwork is strongly correlated to the proportion estimation of CD8 T cells, CD4 memory activated T cells, regulatory T cells (additional file 5).

The “Chemokine-mediated signaling” subnetwork (Fig. 3b) contains 29 genes and three miRNAs. Most of the genes involved in this subnetwork are overexpressed (padj < 0.05). These genes mainly include various cytokines, specifically chemokine ligands and receptors such as *CCL5*, *CCR4*, *CXCL10*, and *XCL1*. Many of these chemokines are involved in calcium signaling, which has been hypothesized by Johari et al. to play a role in IBM [14].

The “Immune response - signal transduction” subnetwork (Fig. 3c) shares several genes with the previous two subnetworks, though the overlap is below the merging threshold. It shares *LCK*, *CD2*, *CD8A*, *UBC*, and *PTPRC* with the “antigen processing and presentation” subnetwork and *CD4*, *UBC*, and *SYK* with the “chemokine-mediated signaling” subnetwork, connecting to these two subnetworks. Only the *CD247*, *ZAP70*, *PIK3R1*, *VAV1*, *STAT1*, *CD28*, *FYN*, *LCP2* and *RACK1* genes are unique to this subnetwork. Genes such as *LCK*, *FYN*,* ZAP70* and *VAV1* are part of T cell receptor signaling [33], which is triggered by binding of the T cell receptor to antigen presenting MHC complexes [34].

The “rRNA processing” subnetwork (Fig. 3d) contains 15 genes. In this subnetwork, the *RPS18* (ribosomal protein S18) gene has a significant variant burden (padj = 0.046) but, interestingly, is not significantly differentially expressed (padj = 0.22). The rest of the genes in this subnetwork are downregulated (padj < 0.05) with no significant variant burden. These include genes like *WDR43*, *IMP4*, *NOP14*, *RRP9*, *PDCD11*, *UTP18*, and *UTP15*, which have a role in the processing of the 18 S ribosomal RNA [35–41].

Finally, the last subnetwork is annotated with “mRNA splicing” (Fig. 3e). Although five of the 38 genes in this subnetwork (*ASPN*, *LRRC17*, *PPIC*, *DOCK4*, and *TLR3*) are overexpressed (padj < 0.05), the rest of the genes are underexpressed (padj < 0.05). Many of these genes, such as *DDX21*, *RBM8A*, *CWC25* and *EFTUD2* are involved in mRNA splicing [42–45]. This subnetwork also contains genes with a significant variant burden, namely *SNAPC4* and *DOCK4*.

## Discussion

This work presents the identification of multi-omics signatures in IBM that provide insights into potential disease mechanisms that are at play. We identified five subnetworks that represent these signatures. Within these subnetworks, we find several interesting interactions which can be the basis for forming hypotheses in IBM.

Many of our results correspond to earlier findings. For example, the HLA genes in the “Antigen processing and presentation” subnetwork are consistently found to be overexpressed in IBM [14, 31]. *LCK* was another overexpressed gene in this subnetwork, previously proposed as an apoptosis regulator involved in IBM [14].

There are also novel findings, such as the miR-16-5p. This miRNA was connected with many HLA genes in the “Antigen processing and presentation” subnetwork. Here, the downregulation of miR-16-5p was negatively correlated with the upregulation of the HLA genes, which is coherent with the canonical mechanism of miRNAs suppressing gene expression. Interestingly, the overexpression of *miR-16* and *miR-15a* led to a significantly decreased pro-inflammatory signaling through IL-1β, TNFα, and NF-κB in a study in mice [46]. In addition, another study in mice links the conditional deletion of these microRNAs to proliferation of T regulatory cells (of which the estimated abundance is correlated to several of our subnetworks) and loss of immune tolerance [47]. At the same time, in an epithelial-like cell line, transfection of *miR-16* led to upregulation of *HLA-G*, *HLA-A*, *HLA-B*, and *HLA-C* [48]. We speculate that *miR-16* could play a role in regulating MHC class I gene transcription in IBM, which in turn regulates immune system activation. Recently, Lucchini et al. identified dysregulation of hsa-miR-192-5p and hsa-miR-372-3p in serum of IBM patients [49]. Furthermore, miR-16 specifically was implicated in several inflammatory diseases including rheumatoid arthritis, ankylosing Spondylitis and inflammatory bowel disease [50–53]. These findings highlight the importance of studying the role of miRNAs in the context of molecular pathomechanisms of IBM. Interestingly, miR-16 is also connected to *SQSTM1*, of which the encoded protein is aggregated in IBM and other myopathies [54, 55]. Despite *SQSTM1* being a proposed target of *miR-16* [49], its expression is not negatively correlated with the *miR-16* in our data,perhaps due the influence of other regulatory factors. *SQSTM1* was also underexpressed as it was in previous studies with RNA-Seq [55].

The “Chemokine-mediated signaling” subnetwork is interesting because many chemokines are involved in calcium signaling. Disturbed calcium signaling has been proposed as a candidate mechanism in IBM [14, 57]. The prominence of this signature in our results (many strongly dysregulated genes that are interconnected) supports this.

Another gene of interest is *RPS18*, which encodes a ribosomal protein. Interestingly, some of the surrounding genes are involved in processing the 18 S ribosomal RNA, which is essential for ribosome function [58]. Alterations in RNA metabolism have been implicated in IBM [59]. In addition, in a proteomics study, ribosomal and nuclear proteins were overrepresented in rimmed vacuoles in IBM compared to controls [60]. These findings suggest a role of altered protein synthesis in IBM.

Similarly, the “mRNA splicing” subnetwork, which contains many underexpressed genes, points to a potential dysfunction of the spliceosome in IBM, especially since there is evidence for genes that have altered splicing in IBM [14, 61, 62]. Specifically, the *SNAPC4* gene is involved in the transcription of snRNAs that are part of the spliceosome. It has a significant variant burden and thus could play a more causal role in altered splicing. *SNAPC4* is also associated with ankylosing spondylitis, an inflammatory disease that affects the spine, and like IBM, has the MHC implicated in its pathogenesis [63].

Finally, our study demonstrates how the application of active subnetwork identification on multi-omics data can connect findings and interactions in different omics, and thereby provide hypotheses about their interplay, An example of this is the link between miR-16-5p and the HLA genes in the antigen processing and presentation subnetwork as shown in our study. In addition, in the rRNA processing subnetwork, we found the RPS18 gene as having a significant variant burden without exhibiting any significant changes in gene expression. We found this gene because it was linked with many downregulated genes that have functions directly related to RPS18. We speculate that variants in RPS18 may affect the expression of the related genes.

Some limitations of our approach are important to note. Although prior knowledge aids in the analysis of omics data in several ways, prior knowledge is also limited by our current understanding of biology and can be biased towards biological concepts that have been studied more. For example, genes that are studied more, such as genes involved in cancer, have more known interactions and are therefore more likely to be overrepresented in network analysis. However, this limitation is mitigated since in our approach we included experimental data in the network. Another limitation primarily affecting multi-omics analysis is the different number of features in each omics. For example, in our analysis, 15,574 nodes represent protein coding genes in our analysis, but only 1831 nodes represent miRNAs. Consequently, relatively few miRNAs were present in the results, which limits the priority of miRNAs in this study.

Our study also has some limitations in terms of data availability. Although for the expression data, a subset was used with a balanced representation in terms of age and sex, the limited sample size still makes our study more sensitive to sampling error caused by individual variation. Further, the accuracy of the cell type proportion through deconvolution estimation is limited since the used reference cell type expression profile (blood) deviates from the muscle disease. Finally, histopathology could not be directly correlated because of subjectivity in sample classification. In order to make also muscle pathology data available as addendum in future studies, streamlining the biopsy procedures and light microscopic analysis processing is recommended.

In future work, the hypotheses surrounding *miR-16-5p* and the *RPS18* gene in IBM could be further studied to increase understanding of the disease and thereby provide opportunities for treatments. Expanding to other diseases, our network based approach can also be applied in diseases with multi-omics data available in order to gain new insights into the interplay between different omics. Finally, the methodology to apply active subnetwork identification on multi-omics datasets Could also be further improved, for example, by exploring normalization and weighting schemes for the multiple omics layers and data sources.

## Conclusions

In this work, we present an integrative approach that combines experimental multi-omics data and prior knowledge for elucidating the mechanisms that are implicated in IBM. We identified five subnetworks that combine findings from different omics datasets and interactions. For example, the antigen processing and presentation subnetwork links genes with differential gene expression to genes with significant variant burden and miRNAs. Specifically, the underexpressed miR-16-5p was connected to multiple overexpressed HLA genes by negative expression correlation. This connection could potentially play a role in the regulation of the HLA genes.

Similarly, we found the RPS18 gene having both a variant burden and being connected to many underexpressed genes involved in 18 S ribosomal RNA processing. Mutations in this gene could thus affect the expression of the connected genes and play a role in IBM. Moreover, our analytical workflow which was implemented using the common workflow language can be reused for other case studies.

## Electronic supplementary material

Below is the link to the electronic supplementary material.

**Additional file 1**: Differential gene expression. Table with results of differential gene expression analysis.

**Additional file 2**: Differential miRNA expression. Table with results of miRNA expression analysis.

**Additional file 3**: Subnetwork nodes. Table of all nodes in the subnetworks, with their *p*-values.

**Additional file 4**: Subnetwork edges. Table of all edges in the subnetwork, with their score or correlation.

**Additional file 5**: Cell type correlations. Figure with correlations of subnetwork expression to estimated cell type proportions.

## Data Availability

The Workflow and code is available on GitHub (https://github.com/dwijnbergen/IBM_ASI_workflow*)* and WorkflowHub (10.48546/workflowhub.workflow.681.7*).* The Docker image is available on https://hub.docker.com/R/jdwijnbergen/multi-omics_asi, and it’s build requirements are available on Zenodo (10.5281/zenodo.10210364*).* The input data of our workflow is available on Zenodo at 10.5281/zenodo.10411125.
