# Supplementary material for: Multi-omics analysis in inclusion body myositis identifies mir-16 responsible for HLA overexpression
Source: Orphanet J Rare Dis. 2025 Jan 15;20:27. doi: 10.1186/s13023-024-03526-x (PMC11737257; doi:10.1186/s13023-024-03526-x)
Supplement: Supplementary file 5 — Additional file 5: Cell type correlations. Figure with correlations of subnetwork expression to estimated cell type proportions. [file 13023_2024_3526_MOESM5_ESM.pdf]

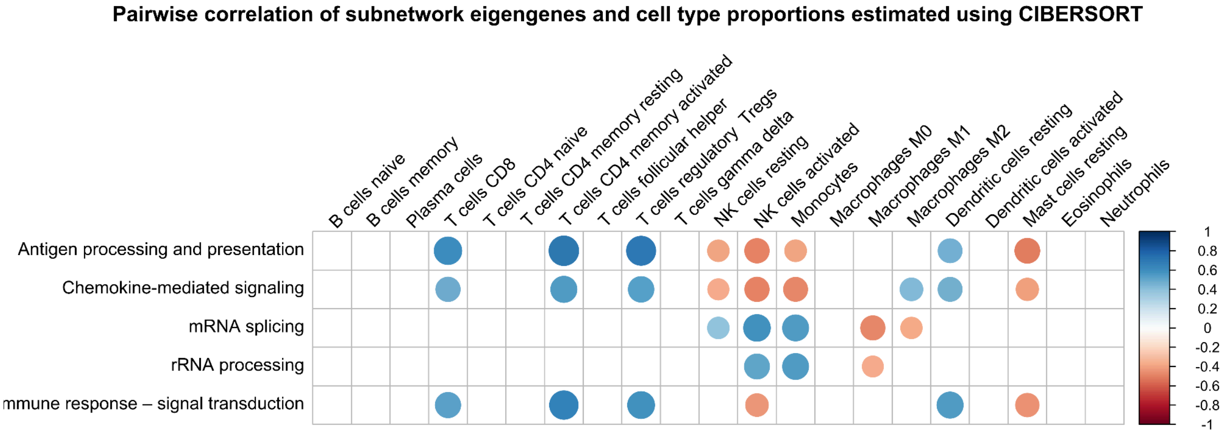

Supplemental figure 1: Pearson correlation of subnetwork eigengenes and estimated cell type proportions. Eigengenes represent the first principal component of each subnetwork. Only correlations with $p < 0.05$ are shown.
